# Identifying symptoms associated with diagnosis of pancreatic exocrine and neuroendocrine neoplasms: a nested case-control study of the UK primary care population

**DOI:** 10.3399/BJGP.2021.0153

**Published:** 2021-09-21

**Authors:** Weiqi Liao, Ashley K Clift, Martina Patone, Carol Coupland, Arturo González-Izquierdo, Stephen P Pereira, Julia Hippisley-Cox

**Affiliations:** Nuffield Department of Primary Care Health Sciences, University of Oxford, Oxford.; Nuffield Department of Primary Care Health Sciences, University of Oxford, Oxford, and Cancer Research UK Oxford Centre, University of Oxford, Oxford.; Nuffield Department of Primary Care Health Sciences, University of Oxford, Oxford.; Division of Primary Care, School of Medicine, University of Nottingham, Nottingham.; UCL Institute of Health Informatics, Health Data Research UK, London.; UCL Institute for Liver and Digestive Health, University College London.; Nuffield Department of Primary Care Health Sciences, University of Oxford, Oxford.

**Keywords:** early diagnosis, pancreatic ductal adenocarcinoma (PDAC), pancreatic neoplasms, pancreatic neuroendocrine neoplasms (PNEN), primary health care, symptom

## Abstract

**Background:**

Pancreatic cancer has the worst survival rate among all cancers. Almost 70% of patients in the UK were diagnosed at Stage IV.

**Aim:**

This study aimed to investigate the symptoms associated with the diagnoses of pancreatic ductal adenocarcinoma (PDAC) and pancreatic neuroendocrine neoplasms (PNEN), and comparatively characterise the symptomatology between the two tumour types to inform earlier diagnosis.

**Design and setting:**

A nested case-control study in primary care was conducted using data from the QResearch^®^ database. Patients aged ≥25 years and diagnosed with PDAC or PNEN during 2000 to 2019 were included as cases. Up to 10 controls from the same general practice were matched with each case by age, sex, and calendar year using incidence density sampling.

**Method:**

Conditional logistic regression was used to investigate the association between the 42 shortlisted symptoms and the diagnoses of PDAC and (or) PNEN in different timeframes relative to the index date, adjusting for patients’ sociodemographic characteristics, lifestyle, and relevant comorbidities.

**Results:**

A total of 23 640 patients were identified as diagnosed with PDAC and 596 with PNEN. Of the symptoms identified, 23 were significantly associated with PDAC, and nine symptoms with PNEN. The two alarm symptoms for both tumours were jaundice and gastrointestinal bleeding. The two newly identified symptoms for PDAC were thirst and dark urine. The risk of unintentional weight loss may be longer than 2 years before the diagnosis of PNEN.

**Conclusion:**

PDAC and PNEN have overlapping symptom profiles. The QCancer^®^ (pancreas) risk prediction model could be updated by including the newly identified symptoms and comorbidities, which could help GPs identify high-risk patients for timely investigation in primary care.

## INTRODUCTION

Pancreatic cancer is the 10th most common cancer in incidence but represents the fifth most common cause of death owing to cancer in the UK. Pancreatic cancer is very aggressive and has the worst survival rate among all types of cancer.^1^ Almost 70% of patients were diagnosed at Stage IV. Tumours arising from the pancreas can be classified as exocrine (approximately 95%) or neuroendocrine (≤5%) neoplasms,^2^ known as pancreatic ductal adenocarcinoma (PDAC) and pancreatic neuroendocrine neoplasms (PNEN),^3^ respectively. Though there are differences in tumour pathobiology and treatment strategies between PDAC and PNEN, the two tumour types share a proclivity to metastasise.^4^^–^6 More favourable outcomes were observed in diagnosis at earlier stages.^7^

In the absence of a screening programme for pancreatic cancer in the UK, symptomatic presentation in general practice remains a key avenue for earlier diagnosis. However, besides jaundice, current literature reporting symptoms associated with pancreatic cancer are vague and non-specific.^8^^,^^9^ GPs face the challenges of differentiating a potential malignancy from other benign diseases when patients present with non-specific symptoms, which could be easily ‘missed’ or delay diagnosis of the tumour. In addition, as a rarer type of cancer, there is still no large-scale study characterising the symptomatology of PNEN, nor have studies comprehensively compared the symptomatology of PDAC with PNEN. Therefore, a better understanding of the symptomatology and the timings at which patients present with symptoms could help GPs better manage patients and make clinical decisions. Furthermore, such symptoms could be used in social campaigns to increase public awareness of pancreatic cancer.

To address this research gap, the authors conducted this study with three aims: to explore the symptoms that patients presented in primary care in different time windows, which may indicate the diagnosis of PDAC/PNEN; to comparatively characterise the symptomatology of PDAC and PNEN; and to inform the update of the QCancer^®^ (pancreas) prediction model.^10^^,^^11^ The QCancer (pancreas) score quantifies the risk of an incident diagnosis of pancreatic cancer in the next 2 years, based on an individual patient’s characteristics. Such a risk score may help GPs make different decisions: a 2-week-wait referral for patients at high risk; watchful wait and safety netting for patients with low risk.

**Table:** How this fits in

This is the largest population-based study of its kind, systematically examining the symptomatology of pancreatic ductal adenocarcinoma (PDAC) and pancreatic neuroendocrine neoplasms (PNEN), and quantifying the association of 42 potential symptoms in different time windows relative to the date of diagnosis. This study confirmed several symptoms as risk factors for PDAC reported in previous UK studies, which had much smaller sample sizes. A deeper understanding of symptoms associated with PNEN is gained. Considering that most symptoms associated with pancreatic cancer are non-specific and do not qualify for urgent referral for investigation (2-week-wait) in the current NICE guideline, GPs should be vigilant of patients presenting with several concurrent non-specific symptoms and make proper safety-netting strategies. GPs should also increase the awareness of the risk of pancreatic cancer among people with comorbidities, and be careful not to attribute potential symptoms of pancreatic cancer to patients with existing health conditions.

## METHOD

### Study design setting

This was a nested case-control study using the QResearch database (version 44), an extensive, validated, anonymised primary care database comprising records of >35 million patients registered in approximately 1500 GP surgeries, spread throughout the UK, which have been using the EMIS System since 1989. The population in the QResearch database is representative of the UK population. Patients’ records have been linked with cancer registries, Office for National Statistics (ONS) mortality records, and Hospital Episode Statistics (HES). The cancer registration data include information on the date of diagnosis, type and location of the tumour, morphology, grade and stage, and treatment.

### Study population

The eligible study population was an open cohort of patients aged ≥25 years and registered in the QResearch database between 1 January 2000 and 31 December 2019. Patients with an existing diagnosis of any type of pancreatic cancer before the entry date were excluded. The entry date to the cohort was the latest of the patient’s 25th birthday, the date of patients registered with the practice plus 1 year, the date on which the practice computer system was installed plus 1 year, or the beginning of the study period. The right censor date was the earliest date of the following: the date of pancreatic cancer diagnosis, the date of death, the date of leaving the practice, or the study end date. Person years were calculated between the study entry date and the right censor date.

### Identification of cases and controls

Cases were patients in the study cohort with an incident diagnosis of PDAC/PNEN, recorded in ≥1 of the four linked sources — GP records, HES, cancer registry, or ONS. The index date for cases was the earliest date the diagnosis was recorded in any four data sources. Cases were matched with up to 10 controls in the same practice, age, sex, and calendar year using incidence density sampling.^12^ Each control was allocated an index date, which was the date of diagnosis of their matched case.

### Candidate symptoms and potential risk factors

A broad list of the 42 symptoms potentially associated with PDAC and PNEN is summarised in Box 1. These symptoms were identified through literature review,^2^^,^^10^^,^^13^^,^^14^ information from the leading charities such as Cancer Research UK^15^ and Pancreatic Cancer UK,^16^ National Institute for Health and Care Excellence (NICE) guidelines — NG12^17^ and NG85,^18^ and patient representatives. All occurrences of symptoms in primary care records were extracted, but the analysis was focused on the most recent 5 years before the index date. In addition, the following variables were of research interest and adjusted in the models: patients’ sociodemographic characteristics (ethnicity, socioeconomic deprivation using Townsend quintile), lifestyle factors (smoking and drinking statuses, and body mass index [BMI], using most recently available values before the index date), and relevant comorbidities that could cause the symptoms, or be potential risk factors for, pancreatic cancer.

**Box 1.** Overview of 42 candidate symptoms examined in study

Symptoms in the QCancer (pancreas) model: abdominal distension (females only), abdominal pain, appetite loss, change in bowel habit, constipation (males only), dysphagia (males only), haematemesis, indigestion, and weight loss Symptoms from the literature: back pain, bloating, diarrhoea, fever/shivering, jaundice, itching/pruritus, nausea, steatorrhoea, and vomiting Other potential symptoms to be examined in association with the diagnosis of PDAC/PNEN: abdominal mass, gastrointestinal bleeding, rectal bleeding, heartburn/gastro-oesophageal reflux (GOR), food regurgitation, flatulence, faecal urgency, dark urine, dry mouth, cracked lips, thirst, loss of taste, night sweats, flushes, rash, paleness, oedema, tiredness, appetite increase, weight gain, bad breath, bruising, sore lips, and hypersomnia

### Statistical analysis

Descriptive statistics were used to summarise the sociodemographic and clinical characteristics of patients diagnosed with PDAC and PNEN, and the matched control group. The key clinical characteristics of PDAC and PNEN cases were compared.

Exploratory analyses were conducted to investigate the association between the most recently recorded symptom relative to the index date in seven different periods and patient groups (case/control) using univariable conditional logistic regression. These seven timeframes were <1 month, 1–3 months, 4–6 months, 7–12 months, 1–2 years, 2–3 years, and 3–5 years before the index date. The purpose of setting different timeframes for the same symptoms was to compare how the odds ratio (OR) would change, and the implication of timeframes for earlier diagnosis of PDAC and PNEN based on symptomatic presentation. Based on the exploratory results and the clinical relevance of timeframes for earlier diagnosis of pancreatic cancer, the seven timeframes were narrowed down to the following four in two sets of analysis:

- within 3 months for alarm symptoms, or 1 year for other symptoms (denoted as 3M/1Y); and
- within 6 months for alarm symptoms, or 2 years for other symptoms (denoted as 6M/2Y).

Alarm symptoms included jaundice, dysphagia, and gastrointestinal (GI) bleeding. These three symptoms were analysed within shorter timeframes (within 3 to 6 months before the index date), as they are widely accepted as ‘red flag’ symptoms and should be promptly investigated in primary care, or referred to secondary care. The timeframes were longer for the other symptoms (within 1 or 2 years before the index date), as they are non-specific, probably caused by other benign conditions, and not easily ascribed to an underlying tumour. A categorical variable was used to denote whether the patients presented to their GPs for each symptom based on the most recent date of presentation, for example, no record of presenting with jaundice (reference category), presenting with jaundice within 3 months, or >3 months before the index date. Symptoms in other timeframes (6 months, 1 or 2 years) were operationalised in the same way.

Because of the large difference in sample sizes, PDAC and PNEN were analysed separately. Five variables contained missing data, including ethnicity, Townsend quintile, BMI, smoking and drinking statuses. Multiple imputation with chained equations was used to impute missing values for these variables under the missing at random assumption. Ten imputations were conducted. Multivariable conditional logistic regression models were used to identify symptoms significantly associated with the diagnoses of PDAC and PNENs, adjusting for patient characteristics and comorbidities, with Rubin’s rules used to pool the parameter estimates across the 10 imputed datasets.^19^ Possible interactions were considered and tested in the model. Odds ratios (OR) and 95% confidence intervals (CI) for each symptom were calculated and visualised in forest plots. Symptoms with an OR >1.2 at a significance level of *P*<0.01 for PDAC or PNEN were considered clinically and statistically relevant. Sensitivity analyses were conducted in patients (both cases and control) with at least 3 years of electronic health records (EHRs) before the index date. All statistical analyses were conducted in Stata (version 16.1). The reporting of this study followed the recommendations of the STROBE (strengthening the reporting of observational studies in epidemiology) statement.^20^

## RESULTS

### Population characteristics

The open cohort included 15 194 279 patients aged ≥25 years, with a total of 100 290 294 person years of follow-up. A total of 23 640 PDAC and 596 PNEN cases were identified from the cohort. Case ascertainment from the linked data sources is shown in Supplementary Table S1. The age-standardised incidence rate of PDAC was 23.21 (95% CI = 22.91 to 23.51) per 100 000 person years and 0.95 (95% CI = 0.89 to 1.01) per 100 000 person years for PNEN (data not shown). Table 1 summarises the demographical and clinical characteristics of the cases, organised by tumour type. The comparison of characteristics between cases and controls (*N* = 230 024) is shown in Table 2.

**Table 1.** Clinicopathological characteristics of study cases with pancreatic cancers^a^

**Characteristics**	**PDAC *n* (%) (*N* = 23 640)**	**PNEN *n* (%) (*N* = 596)**
**Age at diagnosis, years**		
25–29	11 (0.05)	<5
30–39	129 (0.55)	25 (4.19)
40–49	666 (2.82)	84 (14.09)
50–59	2325 (9.84)	118 (19.80)
60–69	5176 (21.89)	177 (29.70)
70–79	7717 (32.64)	135 (22.65)
≥80	7616 (32.22)	54 (9.06)

**Route to diagnosis**		
Death certificate	85 (0.36)	<5
Emergency presentation	5542 (23.44)	78 (13.09)
GP referral	2442 (10.33)	147 (24.66)
Inpatient elective	364 (1.54)	23 (3.86)
Other outpatient pathway	1125 (4.76)	112 (18.79)
2-week-wait	1887 (7.98)	57 (9.56)
Not recorded	12 195 (51.59)	178 (29.87)

**Stage at diagnosis (TNM)**		
Stage I	274 (1.16)	62 (10.40)
Stage II	769 (3.25)	53 (8.89)
Stage III	593 (2.51)	27 (4.53)
Stage IV	4016 (16.99)	161 (27.01)
Not recorded	17 988 (76.09)	293 (49.16)

**Grade at diagnosis**		
Well differentiated	303 (1.28)	230 (38.59)
Moderately differentiated	1683 (7.12)	58 (9.73)
Poorly differentiated	1702 (7.20)	61 (10.23)
Undifferentiated	49 (0.21)	<5 (NA)
Not recorded	19 903 (84.19)	246 (41.28)

**Type of tumour**		
Functional NEN	NA	16 (2.68)
Non-functional NEN	NA	271 (45.47)
Neuroendocrine carcinoma	NA	274 (45.97)
Mixed adenocarcinoma/NEN	NA	8 (1.34)
Other/NOS	NA	23 (3.86)
PDAC	23 640 (100)	NA

**Treatment**		
Surgery		
No	17 026 (72.02)	220 (36.91)
Yes	6614 (27.98)	376 (63.09)
Chemotherapy		
No	19 402 (82.07)	430 (72.15)
Yes	4238 (17.93)	166 (27.85)
Radiotherapy		
No	23 000 (97.29)	557 (93.46)
Yes	640 (2.71)	39 (6.54)
Hormone therapy		
No	23 574 (99.72)	550 (92.28)
Yes	66 (0.28)	46 (7.72)
Other treatment		
No	19 030 (80.50)	458 (76.85)
Yes	4610 (19.50)	138 (23.15)

a *Rows with counts <5 have been suppressed. NA = not applicable. NEN = neuroendocrine neoplasm. NOS = not otherwise specified. PDAC = pancreatic ductal adenocarcinoma. PNEN = pancreatic neuroendocrine neoplasm. TNM = TNM Classification of Malignant Tumours.*

**Table 2.** Demographics, lifestyle, and comorbidities in case controls^a^

**Characteristics**	**Cases with PDAC *n* (%) (*N*= 23 640)**	**Cases with PNEN *n* (%) (*N*= 596)**	**Controls *n* (%) (*N*= 230 024)**
**Age, years**			
Mean (SD)	73 (11.5)	62.3 (13.2)	72.0 (11.4)
Median (IQR)	74 (66–82)	64 (53–72)	73 (65–81)

**Sex**			
Female	11 705 (49.51)	294 (49.33)	114 429 (49.75)
Male	11 935 (50.49)	302 (50.67)	115 595 (50.25)

**Ethnicity**			
White	12 604 (53.32)	367 (61.58)	154 096 (66.99)
Indian	186 (0.79)	9 (1.51)	2674 (1.16)
Pakistani	100 (0.42)	6 (1.01)	1150 (0.50)
Bangladeshi	47 (0.20)	<5	692 (0.30)
Other Asian	79 (0.33)	10 (1.68)	1223 (0.53)
Caribbean	247 (1.04)	10 (1.68)	2364 (1.03)
Black African	103 (0.44)	<5 (NA)	1369 (0.60)
Chinese	36 (0.15)	<5 (NA)	449 (0.20)
Other	166 (0.70)	12 (2.01)	1853 (0.81)
Not recorded	10 072 (42.61)	173 (29.03)	64 154 (27.89)

**Townsend quintile**			
1 (most affluent)	7054 (29.84)	190 (31.88)	72 988 (31.73)
2	5873 (24.84)	135 (22.65)	58 901 (25.61)
3	4758 (20.13)	126 (21.14)	44 581 (19.38)
4	3442 (14.56)	91 (15.27)	31 513 (13.70)
5 (most deprived)	2470 (10.45)	54 (9.06)	21 758 (9.46)
Not recorded	43 (0.18)	0 (0.00)	283 (0.12)

**Year of index date**			
2000–2004	4358 (18.43)	68 (11.41)	42 402 (18.43)
2005–2009	5744 (24.30)	107 (17.95)	55 270 (24.03)
2010–2014	6767 (28.63)	225 (37.75)	66 009 (28.70)
2015–2019	6771 (28.64)	196 (32.89)	66 343 (28.84)

**Smoking status**			
Non-smoker	10 841 (45.86)	327 (54.87)	118 626 (51.57)
Ex-smoker	7117 (30.11)	170 (28.52)	76 842 (33.41)
Light smoker (<10/day)	3047 (12.89)	45 (7.55)	16 917 (7.35)
Moderate smoker (10–19)	617 (2.61)	8 (1.34)	2908 (1.26)
Heavy smoker (≥20)	394 (1.67)	7 (1.17)	1959 (0.85)
Not recorded	1624 (6.87)	39 (6.54)	12 772 (5.55)

**Alcohol consumption**			
Non-drinker	14 053 (59.45)	354 (59.40)	139 902 (60.82)
Trivial (<1 units/day)	3224 (13.64)	89 (14.93)	32 028 (13.92)
Light (1–2 units/day)	1480 (6.26)	41 (6.88)	15 194 (6.61)
Moderate (3–6 units/day)	1539 (6.51)	32 (5.37)	14 712 (6.40)
Heavy (7–9 units/day)	142 (0.60)	<5 (NA)	1173 (0.51)
Very heavy (>9 units/day)	67 (0.28)	<5 (NA)	549 (0.24)
Not recorded	3135 (13.26)	75 (12.58)	26 466 (11.51)

**Comorbidities/previous medical history**			
Type 1 diabetes	534 (2.26)	25 (4.19)	1752 (0.76)
Type 2 diabetes	6581 (27.84)	187 (31.38)	38 666 (16.81)
Venous thrombus embolism	2631 (11.13)	62 (10.40)	12 565 (5.46)
Deep vein thrombosis	1632 (6.90)	35 (5.87)	8246 (3.58)
Pulmonary embolism	1320 (5.58)	34 (5.70)	5640 (2.45)
Acute pancreatitis	857 (3.63)	21 (3.52)	2208 (0.96)
Chronic pancreatitis	477 (2.02)	6 (1.01)	409 (0.18)
Cholangitis	383 (1.62)	8 (1.34)	703 (0.31)
Gallstones	1602 (6.78)	44 (7.38)	12 378 (5.38)
*H. pylori* infection	996 (4.21)	37 (6.21)	8359 (3.63)
Family history of GI cancer	456 (1.93)	15 (2.52)	4074 (1.77)
Blood cancer	350 (1.48)	21 (3.52)	4123 (1.79)
Colon cancer	371 (1.57)	10 (1.68)	3829 (1.66)

a *Rows with counts <5 have been suppressed. GI = gastrointestinal. IQR = interquartile range. PDAC = pancreatic ductal adenocarcinoma. PNEN = pancreatic neuroendocrine neoplasm. SD = standard deviation.*

### Symptoms associated with PDAC and PNEN

Figures 1a and 1b present the significant results of multivariable analyses for PDAC and PNEN for symptoms recorded within 3 months (alarm symptoms) or 1 year (nonspecific) before the index date, respectively, adjusting for patient characteristics and comorbidities. The significant results for symptoms in the 6M/2Y timeframe for PDAC and PNEN are presented in Figures 2a and 2b (only significant results have been plotted, but full results are available in Supplementary Tables S2–S5). Most results were congruent in the two models, though symptoms in a shorter timeframe (3M/1Y) generally had higher ORs than those in a longer period (6M/2Y) and had wider confidence intervals. In addition, symptoms within the cut-off periods (for example, 3 to 6 months or 1 to 2 years) were statistically significant. For symptoms longer than the cut-off periods, there were two main patterns: either the symptoms became non-significant, or the direction of OR in the symptoms reversed (from >1 to <1, significantly higher odds in control), which meant the controls were more likely to consult with those (non-specific) symptoms after the cut-off periods.

**Figure 1a.**
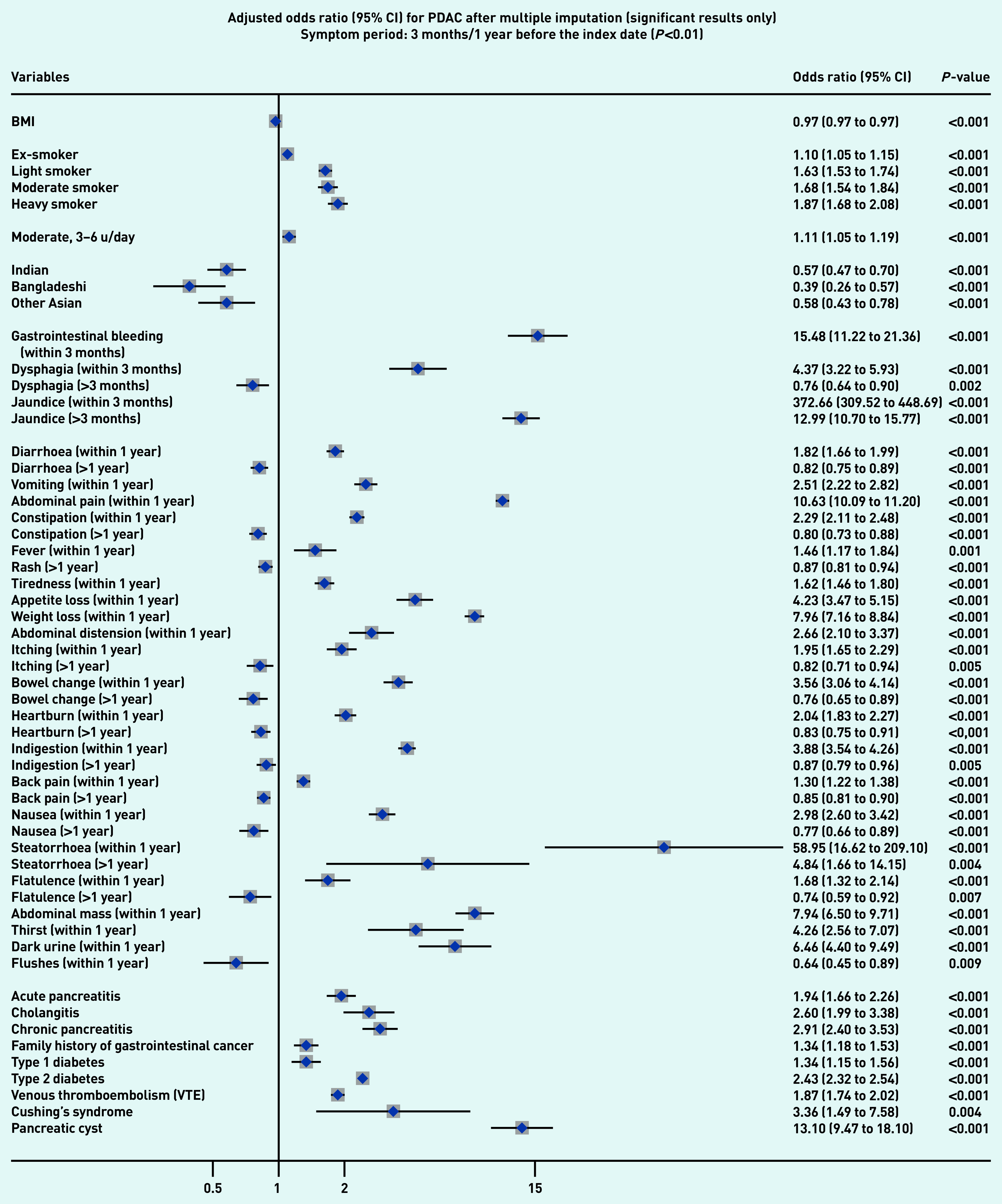
*Forest plots for multivariable conditional logistic regression after multiple imputation (alarm symptoms 3 months before the index date, other symptoms 1 year before the index date) for PDAC. Because of a large number of variables in the model, only significant results (adjusted odds ratio and 95% CI) are presented in the figures. Full results of the models are available in Supplementary Tables S2–S3. The cut-off period for jaundice, gastrointestinal bleeding, and dysphagia was 3 months; 1 year for other symptoms. BMI = body mass index. CI = confidence interval. PDAC = pancreatic ductal adenocarcinoma. u = units.*

**Figure 1b.**
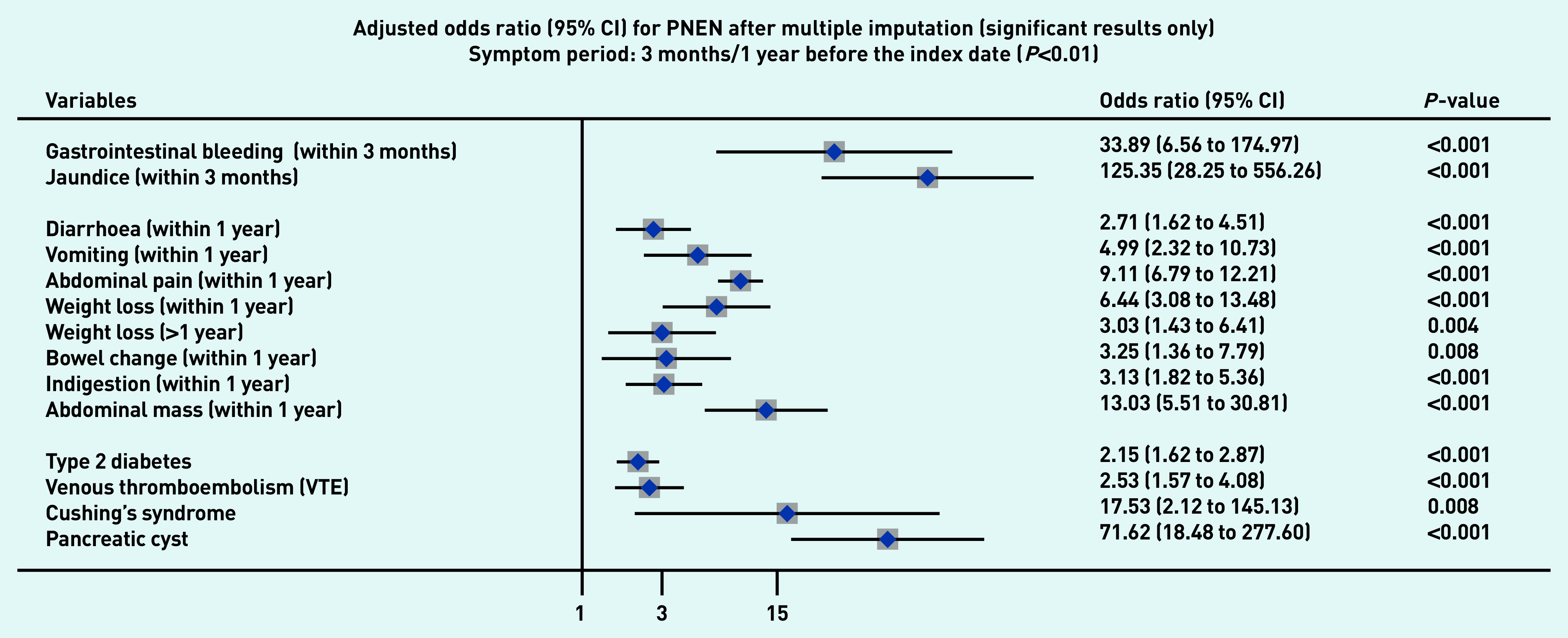
*Forest plots for multivariable conditional logistic regression after multiple imputation (alarm symptoms 3 months before the index date, other symptoms 1 year before the index date) for PNEN. Because of a large number of variables in the model, only significant results (adjusted odds ratio and 95% CI) are presented in the figures. Full results of the models are available in Supplementary Tables S2–S3. The cutoff period for jaundice, gastrointestinal bleeding, and dysphagia was 3 months; 1 year for other symptoms. BMI = body mass index. CI = confidence interval. PNEN = pancreatic neuroendocrine neoplasm.*

**Figure 2a.**
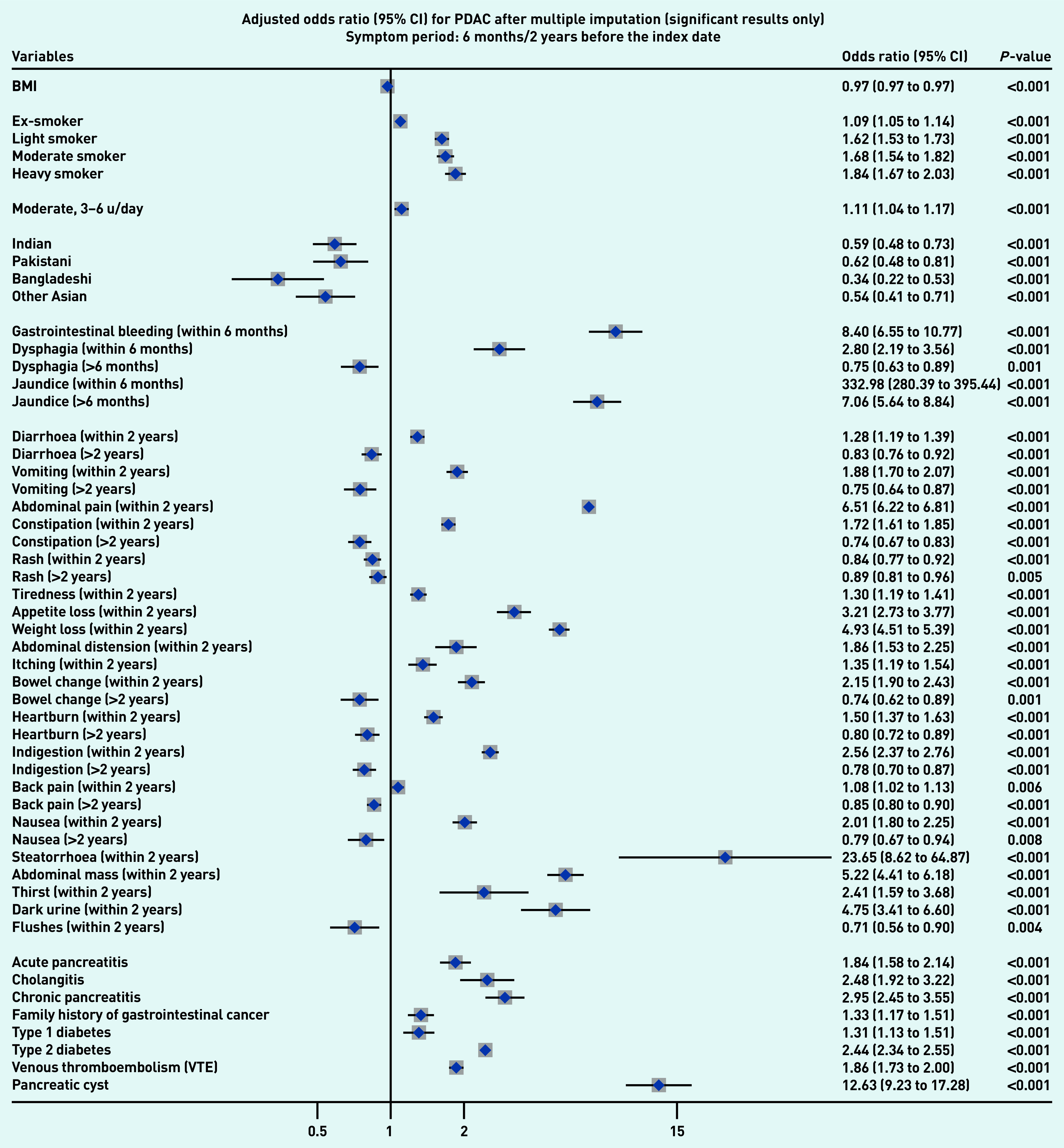
*Forest plots for multivariable conditional logistic regression after multiple imputation (alarm symptoms 6 months before the index date, other symptoms 2 years before the index date) for PDAC. Because of a large number of variables in the model, only significant results (adjusted odds ratio and 95% CI) were plotted in the figures. Full results of the models are available in Supplementary Tables S4–S5. The cutoff period for jaundice, gastrointestinal bleeding, and dysphagia was 6 months; 2 years for other symptoms. BMI = body mass index. CI = confidence interval. PDAC = pancreatic ductal adenocarcinoma. u = units.*

**Figure 2b.**
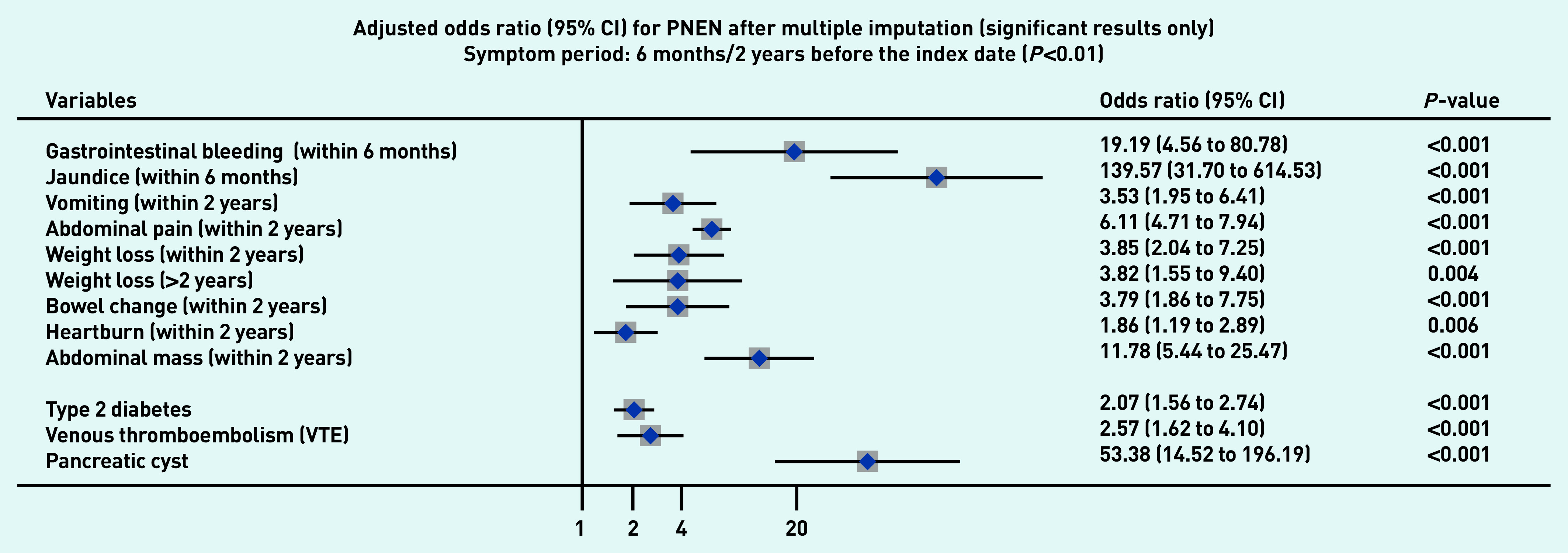
*Forest plot for multivariable conditional logistic regression after multiple imputation (alarm symptoms 6 months before the index date, other symptoms 2 years before the index date) for PNEN. Because of a large number of variables in the model, only significant results (adjusted odds ratio and 95% CI) were plotted in the figures. Full results of the models are available in Supplementary Tables S4–S5. The cut-off period for jaundice, gastrointestinal bleeding, and dysphagia was 6 months; 2 years for other symptoms. BMI = body mass index. CI = confidence interval. PDAC = pancreatic ductal adenocarcinoma. PNEN = pancreatic neuroendocrine neoplasm.*

Noticeably, the effect of unintentional weight loss may be longer than 2 years before the diagnosis of PNEN. Interaction terms were tested; however, they were not in the final model because including interaction terms increased the number of parameters that need to be estimated. The model was unable to converge, especially for PNEN with a small sample size.

Jaundice had the highest adjusted OR in both PDAC and PNEN. Symptoms associated with PNEN were a subset of symptoms associated with PDAC, but the strength of ORs among the significant symptoms may not be the same in PDAC and PNEN. Nine symptoms were significantly associated with the diagnosis of PNEN in the timeframe of 3M/1Y, including jaundice, GI bleeding, diarrhoea, bowel change, vomiting, indigestion, abdominal mass, abdominal pain, and weight loss. The additional significant symptoms associated with PDAC included constipation, steatorrhea, abdominal distension, nausea, flatulence, heartburn, fever, tiredness, appetite loss, itching, back pain, thirst, and dark urine (Figure 1a).

### Other risk factors

Compared with people of white ethnicity, people of Indian, Bangladeshi, and other Asian (not including Chinese) ethnicity were less likely to develop PDAC (OR<1). Smoking and drinking were risk factors for PDAC. When considering comorbidities, type 2 diabetes mellitus (T2DM), venous thromboembolism, Cushing’s syndrome, and presence of pancreatic cysts significantly increased the risks of PDAC and PNEN. Acute pancreatitis, cholangitis, a family history of GI cancer, and type 1 diabetes were significant risk factors for PDAC, but not for PNEN (Figures 1 and 2, comparing a and b).

### Sensitivity analysis

About 26% (25.9%) of patients (65 884 out of 254 260, including cases and controls) were excluded from the sensitivity analysis. By comparing the results from the main analysis, the conclusion of symptoms in the sensitivity analysis did not change, though there were some changes in OR. Complete results of sensitivity analyses are available in Supplementary Tables S6–S9.

## DISCUSSION

### Summary

A total of 24 236 patients diagnosed with pancreatic cancer during 2000 to 2019 were identified from the QResearch database. Nine symptoms were significantly associated with the diagnosis of PNEN, which is a subset of 23 significant symptoms for PDAC. A shorter timeframe (3M/1Y)) was considered better than a longer one (6M/2Y) for earlier cancer diagnosis, as cases had higher odds of presenting symptoms in the 3M/1Y timeframe. Jaundice had the highest adjusted OR in both PDAC and PNEN. Thirst and dark urine were the two newly identified symptoms associated with PDAC, not previously reported in other studies. Thirst could be a symptom explained by T2DM, which is associated with pancreatic cancer. Dark urine could be caused by progressing liver dysfunction, or the manifestation of biliary duct obstruction. The complete findings of this study are summarised in Box 2.

**Box 2.** Summary of patient and clinical characteristics significantly associated with the diagnosis of PDAC and PNEN

	**PDAC**	**PNEN**
**Demographics**	Reduced risks in Indian, Bangladeshi, other Asian (not including Chinese) compared with white ethnicity (OR <1)	

**Lifestyle**	BMI (OR<1)	
	All smoking categories associated with increased risks compared with non-smokers	
	Moderate drinking (3–6 units/day)	

**Symptoms (within 3 months)**	GI bleeding, jaundice, dysphagia	GI bleeding, jaundice

**Symptoms (within 1 year)**	Diarrhoea, bowel change, vomiting, indigestion, abdominal mass, abdominal pain, weight loss^7^	
	Constipation, steatorrhea, abdominal distension, nausea, flatulence, heartburn, fever, tiredness, appetite loss, itching, back pain, thirst, dark urine	Diarrhoea, bowel change, vomiting, indigestion, abdominal mass, abdominal pain, weight loss (longer than 2 years)

**Comorbidities**	Type 2 diabetes, venous thromboembolism (VTE), Cushing’s syndrome, pancreatic cyst, acute pancreatitis, cholangitis, family history of GI cancer, type 1 diabetes	Type 2 diabetes, VTE, Cushing’s syndrome, pancreatic cyst

*BMI = body mass index. GI = gastrointestinal. OR = odds ratio. PDAC = pancreatic ductal adenocarcinoma. PNEN = pancreatic neuroendocrine neoplasm.*

Early diagnosis of pancreatic cancer from primary care is still challenging, owing to non-specific symptoms. This study identified 23 symptoms associated with the diagnosis of PDAC and nine symptoms for PNEN. Risk prediction models incorporating comprehensive symptomatology would help identify patients with a high risk of developing pancreatic tumours from primary care. Patients could benefit from an earlier cancer diagnosis and better survival outcomes, which can also save costs for the NHS.

### Strengths and limitations

The QResearch database provided rich data for this study, which is by far the largest study of its kind. The representative patient population makes the study findings more generalisable to a broader UK population. The use of EHRs avoided selection, recall, and responder biases from the survey, and also provided benefits from the accuracy of coding and data completeness in the UK general practice. The authors explored the effects of symptoms in seven timeframes first, and then narrowed down to 3M/1Y and 6M/2Y before the index date. Symptoms recorded longer than the timeframes (>3M/1Y, >6M/2Y) were not mixed with no symptom recorded, which provided new information about the symptoms beyond the cut-off periods in cases and controls. Though symptoms were the focus of this study, the background risk factors and relevant comorbidities were taken into account, included, and adjusted for in the model, which is another strength. The authors conducted the study as transparently and thoroughly as possible. The research protocol has been published on the QResearch website. The reporting of this article complies with the STROBE statement.

Information bias in EHRs is the first limitation. The authors could not evaluate how accurately the information was recorded across practices. The recording habit may have considerably differed among GPs. The heterogeneity of recording habits was mitigated by using all possible Read codes for each variable. Because of the small sample size in PNEN cases, it is possible that the full burden of symptoms in PNEN could not be captured. Therefore, the authors could not discern whether a lower number of significant symptoms for PNEN is a lack of statistical power, or PNEN has truly less prominent symptomatology, or both. The researchers planned to explore whether there was any symptom associated with early/late stages at diagnosis in PDAC and PNEN. Unfortunately, the large amount of missing data in cancer staging (Supplementary Table S10) did not allow them to conduct such an analysis.

### Comparison with existing literature

The current study has some improvements from the authors’ previous QCancer (pancreas) prediction model,^10^ including a longer study period, a larger sample size of incident cases, more symptoms examined, and the exploration of PNEN, which resulted in an additional 13 significant symptoms identified. Some UK studies examined the symptomatology of PDAC in primary care settings, with a similar study design (matched case-control study) and statistical method (conditional logistic regression), using the GPRD^21^ and the Health Improvement Network (THIN)^22^ databases. The findings in this study are generally consistent with these two publications. No studies have systematically and robustly evaluated the symptomatology of PNEN in primary care. Patients with PNEN (*n* = 64) reported their symptoms in a voluntary, internet-based survey.

Given the small sample size and potential recall bias in that study,^23^ the authors believe that the present population-based approach offers a more robust and generalisable insight of PNEN symptomatology. Older age, smoking, excess alcohol intake, chronic pancreatitis, and T2DM are common risk factors for pancreatic cancer.^24^^–^28 The findings are the same in the present study.

### Implications for research and practice

Most symptoms identified in this study do not qualify for a rapid referral in the current NICE guideline for suspected (pancreatic) cancer pathway referral (NG12).^17^ GPs should be vigilant to patients presenting with alarm symptoms and non-specific but concerning symptoms, especially when patients have existing comorbidities. Public and patient engagement events could raise public awareness of the symptoms of pancreatic cancer, which may help patients see their GPs more promptly when noticing bodily changes.

Based on the study findings, the authors can update the QCancer (pancreas) prediction model^10^ and develop a new model for PNEN. It is also possible to quantify the risk of patients presenting with several concurrent non-specific symptoms, and the predictive values of such symptom combinations. It would be interesting to further understand how GPs managed and investigated patients presenting with different symptom combinations, and the association with the route to diagnosis and cancer stage.

In addition, there is an ongoing project in the authors’ team, investigating diabetes as a risk pathway towards pancreatic cancer: *Diabetes as a risk pathway towards early diagnosis and prognostication of pancreatic cancer* (www.qresearch.org/research/approved-research-programs-and-projects/diabetes-as-a-risk-pathway-towards-early-diagnosisand-prognostication-of-pancreatic-cancer/).
